# GATA2 deficiency in children and adults with severe pulmonary alveolar proteinosis and hematologic disorders

**DOI:** 10.1186/s12890-015-0083-2

**Published:** 2015-08-12

**Authors:** Matthias Griese, Ralf Zarbock, Ulrich Costabel, Jenna Hildebrandt, Dirk Theegarten, Michael Albert, Antonia Thiel, Andrea Schams, Joanna Lange, Katazyrna Krenke, Traudl Wesselak, Carola Schön, Matthias Kappler, Helmut Blum, Stefan Krebs, Andreas Jung, Carolin Kröner, Christoph Klein, Ilaria Campo, Maurizio Luisetti, Francesco Bonella

**Affiliations:** Hauner Children’s University Hospital, Ludwig-Maximilians University, Member of the German Center for Lung Research, Lindwurmstr. 4, 80337 Munich, Germany; Interstitial and Rare Lung Disease Unit, Ruhrland Hospital, University of Duisburg-Essen, Essen, Germany; Institute for Pathology and Neuropathology, University Hospital Essen, Essen, Germany; Department of Pediatric Pneumology and Allergy, University Hospital, University of Warsaw, Warsaw, Poland; LAFUGA Genomics, Gene center, Ludwig-Maximilians University Munich, Munich, Germany; Institute of Pathology, Ludwig-Maximilians University, Munich, Germany; Department of Molecular Medicine, Pneumology Unit, IRCCS San Matteo Hospital Foundation, University of Pavia, Pavia, Italy

## Abstract

**Background:**

The majority of cases with severe pulmonary alveolar proteinosis (PAP) are caused by auto-antibodies against GM-CSF. A multitude of genetic and exogenous causes are responsible for few other cases. Goal of this study was to determine the prevalence of GATA2 deficiency in children and adults with PAP and hematologic disorders.

**Methods:**

Of 21 patients with GM-CSF-autoantibody negative PAP, 13 had no other organ involvement and 8 had some form of hematologic disorder. The latter were sequenced for GATA2.

**Results:**

Age at start of PAP ranged from 0.3 to 64 years, 4 patients were children. In half of the subjects GATA2-sequence variations were found, two of which were considered disease causing. Those two patients had the typical phenotype of GATA2 deficiency, one of whom additionally showed a previously undescribed feature – a cholesterol pneumonia. Hematologic disorders included chronic myeloic leukemia, juvenile myelo-monocytic leukemia, lymphoblastic leukemia, sideroblastic anemia and two cases of myelodysplastic syndrome (MDS). A 4 year old child with MDS and DiGeorge Syndrome Type 2 was rescued with repetitive whole lung lavages and her PAP was cured with heterologous stem cell transplant.

**Conclusions:**

In children and adults with severe GM-CSF negative PAP a close cooperation between pneumologists and hemato-oncologists is needed to diagnose the underlying diseases, some of which are caused by mutations of transcription factor GATA2. Treatment with whole lung lavages as well as stem cell transplant may be successful.

## Background

Pulmonary alveolar proteinosis (PAP) is a rare disorder characterized by the progressive accumulation of surfactant in the alveoli of the lungs, leading to hypoxemic respiratory failure and, in severe cases, to death [1]. PAP is caused by (i) genetic diseases which result in dysfunction of surfactant or surfactant production (SFTPC, SFTPB, ABCA3, TTF1 deficiency) mainly presenting during infancy, by (ii) disruption of GM-CSF signaling from mutations in the receptor (GM-CSFRa, GM-CSFRb) or from acquired autoantibodies against GM-CSF, and by (iii) disorders that presumably impair surfactant clearance because of abnormal numbers or defective phagocytic functions of alveolar macrophages [2]. The latter are caused by inhaled particles or by hematologic disorders affecting macrophage precursors.

A broad spectrum of hematologic disorders have been associated with PAP, most frequently myelodysplastic syndrome (MDS), and more rarely acute (AML) and chronic myeloid leukaemia (CML), myelofibrosis, acute lymphoid leukaemia (ALL), adult T-cell leukaemia, aplastic anemia, lymphoma, multiple myeloma, plasmacytoma, essential thrombocytosis [3], congenital dyserythropoietic anemia [4], and status after unrelated stem cell transplant [5, 6]. Up to now only mutations in one specific gene -GATA2- have been associated with this form of PAP.

GATA2 is a zinc finger transcription factor essential for differentiation of immature hematopoietic cells [7]. Among many other functions GATA2 regulates the phagocytosis of alveolar macrophages [8]. Alveolar macrophages treated with the sense GATA-2 expression construct show an increase in their phagocytic activity by up to 280 % compared to the antisense construct [9]. GATA2 deficiency is a recently described disorder of hematopoiesis, lymphatics, and immunity, caused by heterozygous mutations leading to haplo-insufficiency of the transcription factor GATA2. The disease presents with a complex array of diagnoses and symptoms of varying extent including MDS, AML, chronic myelomonocytic leukemia (CMML), severe viral, disseminated mycobacterial and invasive fungal infections, pulmonary arterial hypertension, warts, panniculitis, human papillomavirus (HPV) positive tumors, Epstein-Barr virus (EBV) positive tumors, venous thrombosis, lymphedema, sensorineural hearing loss, miscarriage and hypothyroidism [6]. PAP has been reported in about 18 % of all subjects with GATA2 deficiency [6, 10]. However, not much is known on the severity and clinical course of PAP in these subjects, which were exclusively adults. Pneumologists treating patients with severe PAP not caused by autoantibodies against GM-CSF frequently do not know the cause of these rare conditions [11].

Here, we investigate a cohort of children and adults with severe and chronic PAP and hematologic disease for the presence of GATA2 mutations. GATA2 mutations were identified in a minority of patients only. One child with a GATA2 variant was successfully treated initially by therapeutic whole lung lavages (WLL) and eventually by stem cell transplant.

## Methods

All subjects with PAP diagnosed based on the characteristic phenotype by lung biopsy or bronchoalveolar lavage (BAL) and CT scan findings were identified from the kids-lung-register (http://www.kinderlungenregister.de/index.php/en/) within the child-EU project (European Register and Biobank on Childhood Interstitial Lung Diseases, European Commission, FP7, GA 305653).

**Fig. 1:**
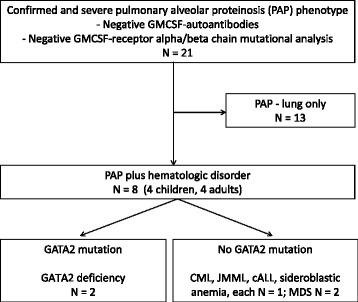
Study design and flow of subjects retrieved from the kids-lung-register data base and biobank. Abbreviations: PAP, pulmonary alveolar proteinosis; CML, chronic myeloic leukemia, JMML, juvenile myelo-monocytic leukemia; cALL, common acute lymphoblastic leukemia; MDS, myelodysplastic syndrome

In order to differentiate subgroups of PAP, all patients with GM-CSF-Ra or Rb mutations [12] and a high GM-CSF autoantibody level were excluded. 21 subjects were identified of whom 13 (11 adults, 2 children) had pulmonary involvement only. 8 subjects suffered from PAP and a hematologic disease - diagnosed by standard procedures- and were selected for further analysis (Fig. 1).

The GATA2 gene was analysed by Sanger sequencing. Genomic DNA was isolated from EDTA blood samples using the QIAamp DNA Blood Mini Kit (Qiagen) and PCR amplified using HotStarTaq polymerase (Qiagen). PCR products were purified with the MinElute 96 UF PCR Purification Kit (Qiagen). Primers used for PCR and sequencing reactions are shown in Table 1. Sequencing was performed by GATC Biotech AG (Konstanz, Germany).

**Table 1:** Sequences of primers used for amplification of the GATA2 gene

Exon	Forward	Reverse
#1	CCC GCA AAG TGA TGT CG	CAA ACG GAC CAA GCG ATT C
#2	ACC TCG TGG TGG GAC TTT G	GAT CCT ACA TCC GGG AAG C
#3/1	GTC CCT AGC TCT GCC TAC CC	CTC CTC GGG CTG CAC TAC
#3/2	ACC TTT TCG GCT TCC CAC	CTC TCC CAA GTC ACA GCT CC
#4	GAC TCC CTC CCG AGA ACT TG	TGT AAT TAA CCG CCA GCT CC
#5	GTG GAG CGA GGG TCA GG	CAC AAA GCG CAG AGG TCC
#6/1	AGG AAT GTT GCT GGA GGA AG	AAC TGT CCA TGC AGG AAA CC
#6/2	GAC ACC ACT CCT GCC AGC	ACA CAG TCA CAG CAG CTT CG
#6/3	TGG AGG GCA GAG ACA ATC AC	AGC AGG GAC ACA GCC TCT C
#6/4	CTT TGC TGC CCT TGG TTT C	AAT CTG GCT GCC CAA ATT C

In subject 4 800 ng of genomic DNA extracted from formalin fixed tissue were fragmented to an average size of 200 bp and desired fragment sizes were obtained with Agencourt® AMPure® XP beads (Beckman Coulter). Sequencing libraries were prepared using Accel-NGS-1S Library Kit (Swift Biosciences, Ann Harbor, MI), sequenced on the HiSeq 1500 (Illumina) and aligned to the human genome assembly (hg19). The GATA2 locus was covered by 86 reads.

Follow-up data of the patients were collected until October 2014. The study was approved by the institutional review board, the ethics commission of the medical faculty of the Ludwig-Maximilians University, Munich, Germany (EK 026–06). All parents or guardians gave their written informed consent, the children gave assent.

## Results

Age at onset of PAP ranged from 0.4 to 64 years. Half of the patients were children (Table 2). Beside chronic respiratory insufficiency due to PAP, all patients suffered from recurrent respiratory tract infections or exacerbations. These were mainly common cold viral infections, in some cases non-tuberculous mycobacteria, herpes simplex virus (HSV), and cytomegalovirus (CMV) were identified. All patients were immunodeficient: in three patients primary immunodeficiency syndromes were identified: two with a monocyte deficiency not further specified (no. 2, 4) and one with a Di George type II syndrome with combined immunodeficiency (no. 7); all three patients are described below in detail. In the other patients, immunodeficiency was secondary to the hematologic condition (CML, juvenile myelomonocytic leukemia (JMML), c-acute lymphoblastic leukemia (cALL), MDS) and/or therapeutic interventions (steroids, immunosuppressant medication).

**Table 2:** Clinical detail of the patients with hematologic PAP

No	Sex	Start pulm. dis. (y)	Course pulm. dis.	Chest CT scan	Treatments	Age at last follow up (y)	Out-come	Final diagnosis and likely cause of PAP
1	F	64	Chronic respiratory insufficiency, PAP, inactive TBC, ARDS	Diffuse ground glass with clear interstitial septal thickening (crazy paving pattern), bilateral alveolar consolidations	Oxygen, WLL (2)	66	dead	CML, karyotype 46XX t(9,22) (q34,q11) 25, CD 2+, renal failure
2	M	34	Respiratory insufficiency, recurrent interstitial pneumonia (HSV); bron-chiolitis obliterans; PAP	Interstitial thickening and destructions in both lungs, crazy-paving pattern	Prednisolone, azathioprine, oxygen, WLL (3)	37	dead	Monocyte defect, *M. avium intracellulare* infection, GATA2 mutation (p.Y377D)
3	M	0.6	Infection, tachydyspnea (7 months old), hypoxia, PAP (1.7 y)	Diffuse ground glass, interstitial markings, emphysema	Oxygen, immune-suppressive drugs, methylprednisolone, WLL (3)	3, lost on follow up	sick-same	Juvenile myelo-monocytic leukemia, Monosomy 7, 2x SCT, intestinal, hepatic, cutaneous GVHD
4	F	38	Dyspnea, clubbing, respiratory insufficiency, cholesterol pneumonia, PAP	Diffuse ground glass, interstitial markings, scattered alveolar opacities	Oxygen, no WLL	43, lost on follow up	sick-same	Monocyte defect, cholesterol pneumonia, GATA2 mutation (p.R398W)
5	F	6	PAP	Ground glass, interstitial pneumonia	Oxygen, no WLL	7	sick-better	C-acute lymphoblastic leukemia
6	F	59	Dyspnea, respiratory insufficiency, PAP	Diffuse ground glass, interstitial septal thickening (crazy paving), markedly basal, with traction bronchiectasis	Oxygen, WLL (1)	59	dead	MDS
7	F	4	Recurrent airway infections; chronic hypoxic failure, PAP	Alveolar opacities in almost all lung areas, see Fig. 2	Oxygen, WLL (14)	7.3	healthy	MDS, DiGeorge Syndrome Type 2, Monosomy 7, Trisomy 8 (SCT)
8	M	0.33	CMV infection, respiratory failure, PAP	Ground glass opacities with interstitial septal thickening (crazy paving pattern), alveolar consolidations bilaterally in the lower parts	Oxygen, WLL (11)	2	sick-better	Sideroblastic anemia

Abbreviations: ARDS, acute respiratory distress syndrome; CML, chronic myeloid leukemia; CMV, cytomegalovirus; dis., disease; GVHD, graft versus host disease; HSV, herpes simplex virus; MDS, myelodysplastic syndrome; PAP, pulmonary alveolar proteinosis; pulm., pulmonary; SCT, stem cell transplant; TB, tuberculosis, WLL, whole lung lavage; y, years; number in (), number of WLL

The hallmark of all patients of this study was severe PAP with continuous need of oxygen (Table 2). All but two subjects (no. 4, 5) were treated symptomatically with repetitive therapeutic WLL to improve respiratory insufficiency. Treatment was eventually not successful in 3 patients who died from respiratory failure, complicated by infection or acute respiratory distress syndrome (ARDS).

Autoantibodies against GM-CSF were determined in order to characterize PAP further, they were negative in all patients (Table 3). Serum levels of GM-CSF were not significantly increased in the patients with serum available. 4 of the 8 patients had sequence anomalies in GATA2 (Table 3). Two of these, a synonymous variant and a missense variant were predicted not to be damaging by Polyphen-2 [13] and SIFT [14]. The other two were heterozygous point mutations predicted to be deleterious, both of these patients had a phenotype characteristic for GATA2 deficiency.

**Table 3:** Laboratory results of the patients with hematologic PAP

No	ID	GM-CSF auto-anti-bodies in serum	GM-CSF in plasma [pg/mL] (normal 5.5 ± 7.2)^a^	GATA2-gene analysis	Interpretation
1	153	Negative	n.a.	c.564 G > C ht; p.T188T	Synonymous variant; C-allele frequency of 0.09
2	163	Negative	5	c.1129 T > G ht; p.Y377D	Missense mutation, deleterious
3	194	Negative	n.a.	Normal	
4	432	Negative	2.9	c.1192C > T ht;p.R398W	Missense mutation, deleterious
5	1505	Negative	0.0	Normal	
6	1740	Negative	17.5	Normal	
7	2334	Negative	11.5	c.490 G > A ht; p.A164T	Missense variant; tolerated, minor allele frequency of 0.24
8	2530	Negative	1.8	Normal	

Abbreviations: *n.a.* not available

^a^Carraway et al. Am J Respir Crit Care Med 2000; 161: 1294–1299

The courses of cases 2, 4, 7 and 8 are reported in more detail to illustrate characteristic presentations.

## Case no. 2

A 34 year old male patient presented with disseminated *Mycobacterium avium intracellulare* infection, including the lungs, chronic labial herpes including a positive serum HSV PCR, and M. Bowen of the skin. Cellular immunodeficiency was suspected based on absent monocytes; the patient was HIV-negative. 3 years later the patient developed respiratory insufficiency, the CT scan showed consolidations suspicious of cryptogenic organizing or atypical pneumonia. Several courses of antibiotic therapy were followed by two pulses of cyclophosphamide; the patient deteriorated further, CT scan showed bilateral interstitial thickening and crazy-paving pattern. Open lung biopsy revealed PAP. Bone marrow aspiration was normal. He was treated symptomatically with WLL under ECMO (extracorporeal membrane oxygenation) support, developed anuric acute renal failure, and died from acute cardiac failure. The patient carried the mutation p.Y377D in the GATA2 gene.

## Case no. 4

A 38 year old female patient presented with recurrent bronchitis and sinusitis, breathlessness during exercise, and interstitial markings on chest x-ray. An open lung biopsy was performed, histology revealed PAP associated with cholesterol pneumonia. Liver and bone marrow biopsies were normal. In the following months she developed progressive peripheral edema and cardiac insufficiency with mild pulmonary hypertension. She further developed a pseudomembranous glomerulonephritis with nephrotic syndrome and a Pseudomonas urosepsis at 39 years. At 40 years cellular immunodeficiency was demonstrated based on the absence of monocytes. She developed condylomatous lesions of the vulva and HPV-associated (types 16 and 28) vulva intraepithelial neoplasia grade 2–3 (VIN2-VIN3), which was resected. Her course was determined by chronic respiratory failure (at rest 4LO2/min; PaO2 45 mmHg (normal > 86), normal PaC02). However, no WLL were performed as the patient was lost on follow up at 43 years. The patient carried the mutation p.R398W in the GATA2 gene.

## Case no. 7

**Fig. 2:**
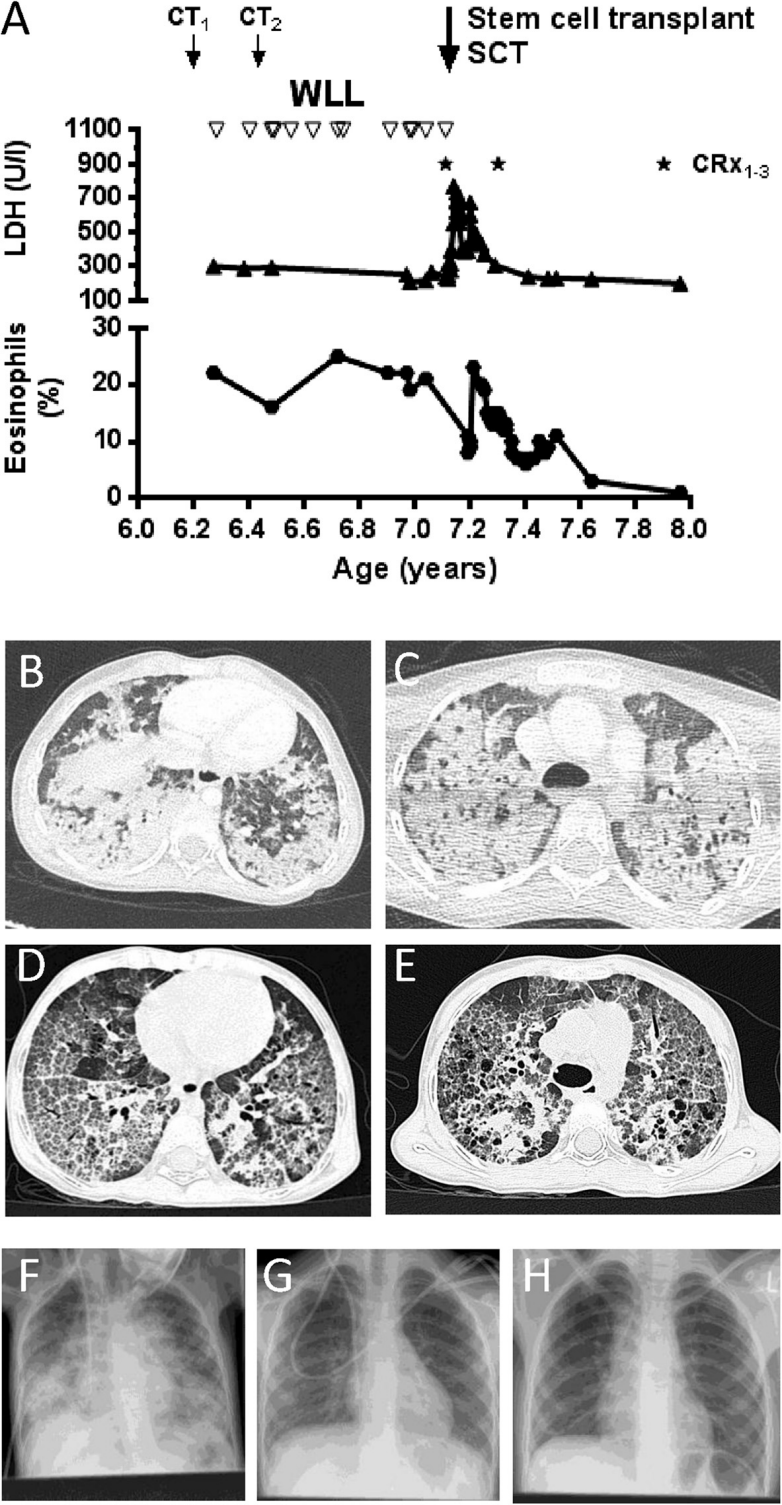
Long term course of a child suffering from DiGeorge syndrome type II and PAP due to MDS with monosomy 7, trisomy 8, and a GATA2 missense variant. Successful treatment by therapeutic WLLs, and definitive treatment of the PAP by SCT. (**a**) clinical course (**b**,**c**) CT1 at presentation (**d**,**e**) CT2 after first 2 whole lung lavages (**f**) CXR 1 before SCT (**g**) CXR2 7 weeks after SCT and (**h**) CXR3 1 year after SCT

A 4.5 year old girl with Di George Syndrome type II from deletion of 10p (OMIM 601362) with severe immunodeficiency, single right kidney, inner ear deafness, mild cognitive delay and dystrophy (BMI 12.0 kg/m2 < 1. percentile)), presented with weight loss, dry cough and frequent night awakenings due to acute dyspnea. The condition deteriorated slowly. She had to sleep in an up-right position when she was referred to our hospital at the age of 6 years. Her oxygen saturation was 75 % without and 92 % with 6 L of O2/min. She had silent breath sounds and a reduced white blood cell count with eosinophilia (1050/μl: neutrophils 8 %, lymphocytes 67 %, monocytes 4 %, eosinophils 19 %, basophils 1 %). MDS with refractory cytopenia (MDS-RC), cytogenetic abnormalities (del 10p, trisomy 8, monosomy 7) and secondary PAP was diagnosed. In BAL fluid elevated eosinophils and granular eosinophilic debris, PAS +, characteristic for PAP were seen. She was treated with 14 WLL by pulmonary artery catheter technique [15], with good clinical and radiological response over a period of one year (Fig. 2). A HLA-identical (10/10) unrelated-stem cell donor was identified at the age of 10 years and she received allogeneic stem cell transplant. Within a few weeks after transplant, PAP and respiratory distress resolved (Fig. 2).

## Case no. 8

A full-term boy with normocytic anemia (Hb 13.1 g/dL) presented after a few weeks with paleness, fatigue, tachydyspnea, and failure to thrive. At the age of four month the hemoglobin level had decreased to 5.3 g/dL and a sideroblastic anemia was diagnosed. No genetic cause was identified; Pearson syndrome and mutations in ALAS2 and SLC25A38 were excluded. His bone marrow showed massive erythroid hyperplasia with dyserythropoiesis and marked depression of myeloid cells. Pulmonary infiltrates were initially interpreted as pneumonia. At the age of 5 months a CMV infection was successfully treated with ganciclovir. At 8 months he developed respiratory failure (PaO2 60 mmHg, PaCO2 55 mmHg) with bilateral pulmonary infiltrates. The diagnosis of PAP was made by analysis of BAL fluid and open lung biopsy. From the age 8 to 23 months the boy underwent 11 therapeutic WLLs, improving his general condition and gas exchange. Currently he maintains oxygen-saturation levels above 92 % under oxygen support 0.5-1 l of O2/min). The anemia is stable with Hb levels between 7.5 and 9.0 g/dL. The molecular cause of very rare sideroblastic anemia was not revealed, the associated depression of myeloid cells was likely the cause of PAP. GATA2 deficiency was not causal, as the patient showed no GATA2 variant in our investigations; potential intronic variations were not investigated.

## Discussion

This study shows that GATA2 mutations in patients with hematologic diseases and severe PAP occur at a relatively low frequency. GATA2 analysis may help to diagnose the underlying hematologic condition. Severe PAP in children due to MDS can be cured by stem cell transplant.

Severe PAP is a rare but serious complication of hematologic disorders. Here we differentiated two groups of diseases having in common a presentation with significant chronic respiratory insufficiency and recurrent pulmonary tract infections or exacerbations. These groups included (i) patients with GATA2 deficiency, a protean disorder of hematopoiesis, lymphatics, and immunity [6], and (ii) patients with functionally or numerically reduced alveolar macrophages and/or their respective mononuclear precursors due to CML, JMML, cALL, MDS or sideroblastic anemia in the absence of disease causing GATA2 mutations.

GATA2 deficiency has a broad phenotype encompassing immunodeficiency, MDS/AML, pulmonary disease, and vascular/lymphatic dysfunction. A precise history and knowledge of the disease course may help to select potential candidates with high confidence for specific genetic diagnostic. GATA2 gene mutations were associated with PAP in two cases; p.Y377D is a novel missense mutation demonstrated here to present with the classical monoMAC syndrome [16]. p.R398W has previously been described in six patients, of whom 2 had a PAP [6]. Here we add the histopathological feature of cholesterol pneumonia to this phenotype. Of interest, further heterozygous variations in GATA2 were identified in two other subjects; however these were predicted to be non-disease causing (Table 3). As not all subjects with a specific GATA2 mutation causing GATA2 deficiency develop PAP [6], additional factors are involved which determine PAP. Patients with the same GATA2 mutation need to be investigated further and experimental models will help to better understand the molecular defect(s) leading to PAP. Similarly, in non-GATA2 deficient patients with PAP and hematologic abnormalities, the mechanism of PAP development is unknown. Currently, their PAP is presumed to be due to reduced monophagocytic function in the alveoli to clear surfactant [17]. Best proof for the critical role of bone-marrow derived alveolar macrophages for surfactant homeostasis is provided by the correction of PAP by SCT. This was shown here for the first time in a young child. Shortly, i.e. within days after take of SCT, there was clearing of PAP, superseding rapidly the need for therapeutic WLLs (Fig. 1).

Here we also show that WLLs are feasible also for children, even at very young age, with small sized airways and in the presence of severe respiratory distress, using techniques we have established previously [18]. WLL can be used to symptomatically treat all types of PAP and to bridge until the underlying condition can be cured or alternative treatments have been implemented.

Due to relative immune deficiency state and irrespective of the cause, the impact of infections should be considered carefully. It is likely that with often severe respiratory tract infections PAP may be triggered to deteriorate. Thus we recommend early diagnosis and proper antimicrobial treatment, in particular of organisms characteristic for these often lethal conditions, including mycobacteria, nocardia, herpes viruses, and fungi [6, 19, 20]. In addition and concordant with the immune deficiency the extensive usage of systemic corticosteroids should be cautioned, as there is so far no evidence of their beneficial effect in PAP. Extensive usage might enhance immune deficiency and weaken antimicrobial defense further [15].

After confirming the diagnosis of PAP either by a combination of characteristic CT scan and BAL findings and trans-bronchial or open lung biopsy, it is important to determine the etiology of PAP. As more than 90 % of PAP in adulthood is caused by autoantibodies against GM-CSF [1, 21, 22], this condition has to be initially excluded by analysis of serum [23, 24]. None of our patients had increased levels of GM-CSF autoantibodies. Serum levels of GM-CSF are low in autoimmune PAP [25], whereas elevated values can be found in congenital GM-CSFRa or GM-CSFRb defects and possibly in other forms of PAP. Thus, particularly in children these other entities need to be excluded. In our cohort of patients with severe PAP and hematologic diseases, serum GM-CSF level were low to intermediate, supporting some up regulation of GM-CSF. Next, genetic analysis of GATA2 is helpful in order to diagnose the underlying hematologic entity more precisely, in particular in patients who meet the broad but characteristic phenotype of GATA2 deficiency [6]. This diagnostic algorithm for PAP will allow differentiating important subgroups and may help to identify further genetic abnormalities, known or suspected to be associated with PAP.

## Conclusions

When investigating a patient with severe PAP, the pneumologist should be aware of the wide range of diseases which can cause secondary PAP and consider interdisciplinary involvement of a hemato-oncologist. Conversely, hemato-oncologists should include PAP in the differential diagnosis of respiratory failure associated with pulmonary interstitial changes in hematologic diseases.

## Abbreviations

ABCA3: ATB-binding cassette sub-family A member 3
ALL: Acute lymphoid leukemia
AML: Acute myeloid leukemia
ARDS: Acute respiratory distress syndrome
BAL: Bronchoalveolar lavage
cALL: common acute lymphoblastic leukemia
CML: Chronic myeloid leukemia
CMML: Chronic myelomonocytic leukemia
CMV: Cytomegalovirus
EBV: Epstein-Barr virus
ECMO: Extracorporeal membrane oxygenation
GM-CSF: Granulocyte macrophage colony-stimulating factor
HPV: Human papilloma virus
HSV: Herpes simplex virus
JMML: Juvenile myelomonocytic leukemia
MDS: Myelodysplastic syndrome
PAP: Pulmonary alveolar proteinosis
SFTPB: Gene encoding for Surfactant protein B
SFTBC: Gene encoding for Surfactant protein C
TTF1: Thyroid transcription factor 1
WLL: Whole lung lavage
